# Real-World Outcomes of Efgartigimod in Adult Myasthenia Gravis

**DOI:** 10.7759/cureus.102031

**Published:** 2026-01-21

**Authors:** Tyler Krall, Samuel Byrne, James Kelbert, Christina Chrisman

**Affiliations:** 1 Neurology, University of Arizona College of Medicine - Phoenix, Phoenix, USA; 2 Neurosurgery, University of Arizona College of Medicine - Phoenix, Phoenix, USA

**Keywords:** clinical outcomes, dosing frequency, efgartigimod, myasthenia gravis, retrospective

## Abstract

Efgartigimod (Vyvgart) is used in the management of myasthenia gravis (MG), but real-world data outside controlled trials, particularly regarding flexible dosing intervals and concomitant medication use, remain limited. The objective of this preliminary, single-center, hypothesis-generating case series was to evaluate the clinical effectiveness of efgartigimod, characterize variability in treatment-free dosing intervals, and explore associations between dosing intervals and concomitant immunosuppressive medication use among adult patients with MG in routine clinical practice.

This was a retrospective chart review of MG patients at a single academic neuromuscular center who were directly evaluated by the principal investigator. Demographics, treatment dates, dosing intervals, concomitant medications, and myasthenia gravis activities of daily living (MG-ADL) scores were collected. Fixed- and mixed-effects models were used to examine associations between efgartigimod use and changes in prednisone or other immunosuppressant dosing.

Nine patients were included (mean age 64 ± 18.1 years, 55.6% male). The average time between cycles was 47 days (SD = 46.5, CI = 34.98-59.01), with wide variability across patients. The average baseline MG-ADL was 8.56 and decreased by 4.44 at 60 days (SD = 3.43), 4.71 at six months (SD = 3.64), and 5.29 at 12 months (SD = 5.06). Reductions were statistically significant at all time points (p = 0.008, 0.016, and 0.031, respectively). Baseline dosing of prednisone, pyridostigmine, and mycophenolate mofetil did not systematically affect cycle length, but higher azathioprine doses were consistently associated with longer subsequent infusion intervals.

Given the small sample size and single-center design, these findings should be interpreted as preliminary observations and hypothesis-generating rather than definitive conclusions.

Efgartigimod was associated with significant improvements in MG-ADLs in this cohort, supporting its effectiveness under flexible, patient-centered dosing schedules. Findings suggest that concomitant azathioprine dosing may be linked to longer infusion intervals, potentially reflecting more stable disease or clinician comfort with extended cycles, although this requires further investigation. Larger, prospective studies are needed to define optimal scheduling strategies, long-term durability, and the potential for reducing polypharmacy.

## Introduction

Overview of myasthenia gravis

Myasthenia gravis (MG) is one of the most common neuromuscular disorders, resulting from autoantibodies to the acetylcholine receptor in the neuromuscular junction [1]. MG is characterized in particular by muscle weakness, worsened with exertion. The most common symptom of MG is oculobulbar weakness and ptosis, followed by dysarthria, dysphagia, and severe weakness of the limbs and diaphragm [2,3]. The prevalence of MG has been estimated to be between 2.19 and 36.71 cases per 100,000 population [2]. This number is expected to increase due to improvements in treatment and life expectancy [3]. Untreated or poorly controlled MG can affect activities of daily living in those affected individuals, and severe disease can be a risk factor for acute myasthenic crisis (MC), which occurs in approximately 15-20% of patients with MG in the first 2-3 years of the disease [4,5].

Pharmacological management of MG 

Pharmacological treatment of MG typically begins with acetylcholinesterase (AchE) inhibitors such as pyridostigmine. However, AchE inhibitors may lead to clinical improvement in only mild forms of MG, yielding the need for additional immunosuppressive therapies [2,4]. Corticosteroids, such as prednisone, are commonly used as a first-line treatment in conjunction with pyridostigmine therapy. If patients fail to respond or require a reduction of their prednisone dose, other immunosuppressive agents may be used as an adjunct medication. Other immunosuppressive drugs with evidence from randomized control trials include azathioprine (AZA), mycophenolate, methotrexate, cyclosporine, and tacrolimus [4]. Ten to fifteen percent of MG cases show no improvement after the introduction of corticosteroids and at least two other immunosuppressive therapies, leading to the need for rescue therapies. These therapies are critical interventions that are aimed at reducing the overall levels of circulating antibodies, leading to an improvement in neuromuscular function [6,7].

Recent FDA-approved MG therapies

Recent limited studies detail clinical outcomes for recent FDA-approved MG therapies. Before 2017, there were no FDA-approved medications for MG; all interventions were prescribed off-label. The FDA has since approved several new therapies, such as eculizumab (2017), efgartigimod (2021), ravulizumab (2022), rozanolixizumab-noli (2023), zilucoplan (2023), and nipocalimab (2025). The mechanisms of MG drugs include complement inhibitors as well as Fc receptor (FcRn) antagonists. Eculizumab, ravulizumab, and zilucoplan are all C5 complement inhibitors that prevent complement-mediated damage from occurring [5,8,9]. Rozanolixizumab-noli, nipocalimab, and efgartigimod are neonatal FcRn antagonists that accelerate the degradation of circulating IgG antibodies [5,9].

Administration varies from intravenous infusion (ravulizumab, eculizumab, nipocalimab, efgartigimod) to subcutaneous infusion (rozanolixizumab-noli) or injection (zilucoplan, efgartigimod) [10-14]. The ADAPT phase 3 trial of efgartigimod showed 68% of AChR-Ab+ patients were MG-ADL responders, versus 30% in placebo [15]. While efficacy and safety are established in trials, real-world effectiveness, side-effect tolerance, and flexible dosing interval management remain underreported [14-18].

Study objectives

Our study is framed as a preliminary case series designed to explore clinical short- and long-term outcomes for adult MG patients on efgartigimod. Short-term and long-term effectiveness were evaluated using within-patient changes in myasthenia gravis activities of daily living (MG-ADL) scores at 60 days, six months, and 12 months following treatment initiation. In addition, the study aimed to characterize variability in treatment-free intervals between efgartigimod cycles and to examine the relationship between baseline concomitant immunosuppressive medication dosing and subsequent infusion intervals.

## Materials and methods

Study design and setting

This study was a retrospective chart review conducted at a single academic neuromuscular center. Given the single-center design and inclusion of only patients evaluated by the principal investigator, this study represents a preliminary case series and may not be fully generalizable to broader MG populations. Institutional review board (IRB) approval was obtained prior to data collection. The electronic medical record (EMR) was reviewed to identify adult patients with a confirmed diagnosis of MG who were treated with efgartigimod. A total of nine patients met eligibility criteria and were included in the final analysis. All extracted data were entered into REDCap, a secure, HIPAA-compliant platform used for research data management [19].

Cohort identification and eligibility criteria

Patients were eligible for inclusion if they were 18 years of age or older, had a clinically confirmed diagnosis of MG, and received at least one treatment cycle of efgartigimod at the study institution. To maintain consistency in clinical evaluation and treatment decision-making, only patients directly evaluated by the principal investigator were included. Exclusion criteria included age younger than 18 years, lack of treatment with efgartigimod, and follow-up conducted exclusively by providers other than the principal investigator.

Data collection and variables

For all included patients, demographic information such as age, sex, and antibody status was collected along with treatment dates and calculated intervals between efgartigimod infusion cycles. MG-ADL scores were recorded at baseline and at 60 days, six months, and 12 months following treatment initiation. Baseline doses of concomitant medications, including prednisone, AZA, mycophenolate mofetil (MMF), and pyridostigmine, were extracted to evaluate whether immunosuppressant or symptomatic therapy dosing influenced subsequent infusion intervals. The MG-ADL scale is a validated, publicly available, and free-to-use outcome measure assessing MG symptom severity across daily functioning domains [20]. Because this was a retrospective review, MG-ADL scores were not consistently collected at identical time points for all patients, which introduces variability and potential measurement bias in the longitudinal assessment.

Outcomes

The primary outcome was the change in MG-ADL scores following initiation of efgartigimod therapy. Secondary outcomes included variability in treatment-free intervals between infusion cycles and potential associations between baseline immunosuppressant dosing and subsequent cycle lengths.

Statistical analysis

All analyses were conducted using the most up-to-date version of RStudio with R version 4.3.1 [21,22]. Changes in MG-ADL scores from baseline to each follow-up interval were evaluated using the Wilcoxon signed-rank test and p-values reported. Treatment interval variability was described using means, standard deviations, and 95% confidence intervals. To examine whether baseline medication dosing predicted the length of subsequent infusion intervals, fixed-effects and mixed-effects linear regression models were constructed, and model outputs included regression coefficients, standard errors, and p-values. Jitter plots and box-and-whisker plots were generated to visualize variability in infusion intervals and MG-ADL trajectories over time.

## Results

Baseline patient characteristics

A total of nine patients diagnosed with MG and being treated with efgartigimod were identified. Baseline demographic and clinical characteristics were summarized (Table 1). The average age of patients was 64 ± 18.1 years, with 5 (55.6%) male patients and 4 (44.4%) female patients. Patients were most commonly White/Caucasian (88.9%), with one patient documented as other (11.1%). The average baseline MG-ADL score was 8.56 ± 4.16, with the highest being 17 and the lowest being 0.

**Table 1:** Demographic characteristics of study participants (N=9). MG-ADL, myasthenia gravis activities of daily living.

Categories	Number	Percentage	Mean	Standard deviation
Age	9		64.3 (37-86)	18.1
Male	5	55.6		
Female	4	44.4		
African American	0	0		
American Indian/Alaskan Native	0	0		
Asian	0	0		
White or Caucasian	8	88.9		
Native Hawaiian or Other Pacific Islander	0	0		
Other	1	11.1		
Hispanic	0	0		
Not Hispanic	8	88.9		
Other	0	0		
Not Specified	1	11.1		
Baseline MG-ADL Score	9		8.56	4.16

Treatment effects, variation in cycle timing, and MG-ADL scores

**Figure 1:**
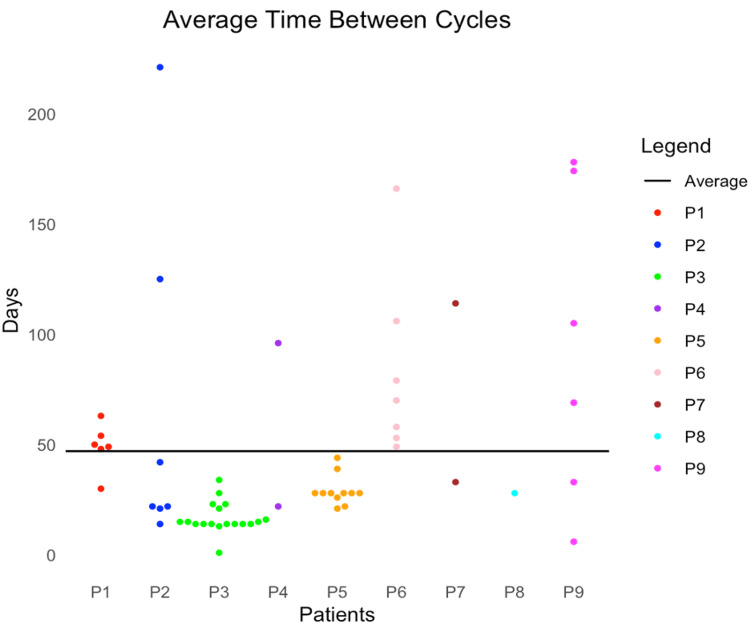
Time between treatment cycles for individual patients as well as the average for the overall study (N = 9).

**Figure 2:**
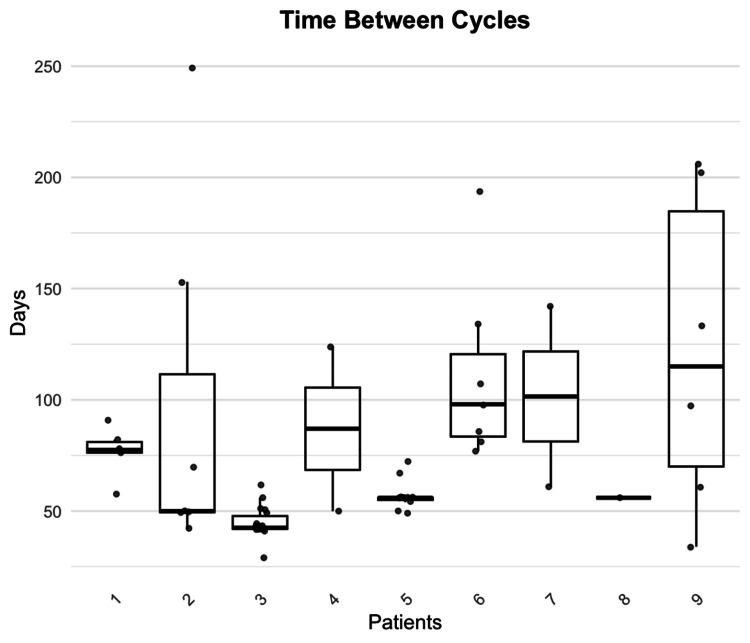
Time between treatment cycles for individual patients (N = 9).

**Figure 3:**
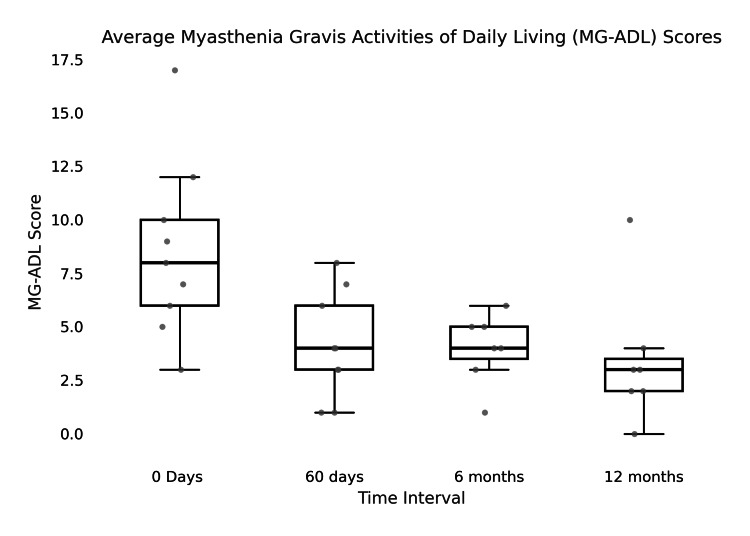
Average MG-ADL scores since initiation of treatment (N = 9). MG-ADL, myasthenia gravis activities of daily living.

The time between cycles significantly varied between all nine patients, with an average of 47 days (SD = 46.5, CI = 34.98-59.01) (Figure 1). Individual patient averages ranged from 44 to 122 days (Figure 2). The average baseline MG-ADL score of 8.56 subsequently decreased over 60 days, six months, and 12 months for patients upon initiation of treatment (Figure 3). The average decrease in MG-ADL score was 4.44 ± 3.43 (51.9%) at 60 days, 4.71± 3.64 (55.0%) at six months, and 5.29 ± 5.06 (61.7%) at 12 months (Table 2). There was a statistically significant reduction in the MG-ADL score at all time intervals (p-value = 0.008 at 60 days, 0.016 at six months, and 0.031 at 12 months).

**Table 2:** Changes in MG-ADL scores from baseline at 60 days, six months, and 12 months after treatment initiation. Values represent the change from baseline MG-ADL score (baseline minus follow-up score), such that positive values indicate improvement (decrease in MG-ADL). Data are reported as mean change, standard deviation (SD), and the number of patients (n) at each time point. Statistical significance of change from baseline was assessed using the Wilcoxon signed-rank test (W = test statistic). MG-ADL scores improved significantly at 60 days (W = 0.00, p = 0.008), six months (W = 0.00, p = 0.016), and 12 months (W = 1.00, p = 0.031) (N = 9). MG-ADL, myasthenia gravis activities of daily living.

Time since start of treatment	Mean MG-ADL change from baseline	Standard deviation	Wilcoxon W	p-Value
60 days	4.44	3.43	0	0.008
6 months	4.71	3.64	0	0.016
12 months	5.29	5.06	1	0.031

Cycle interval variability and concomitant medication use

**Figure 4:**
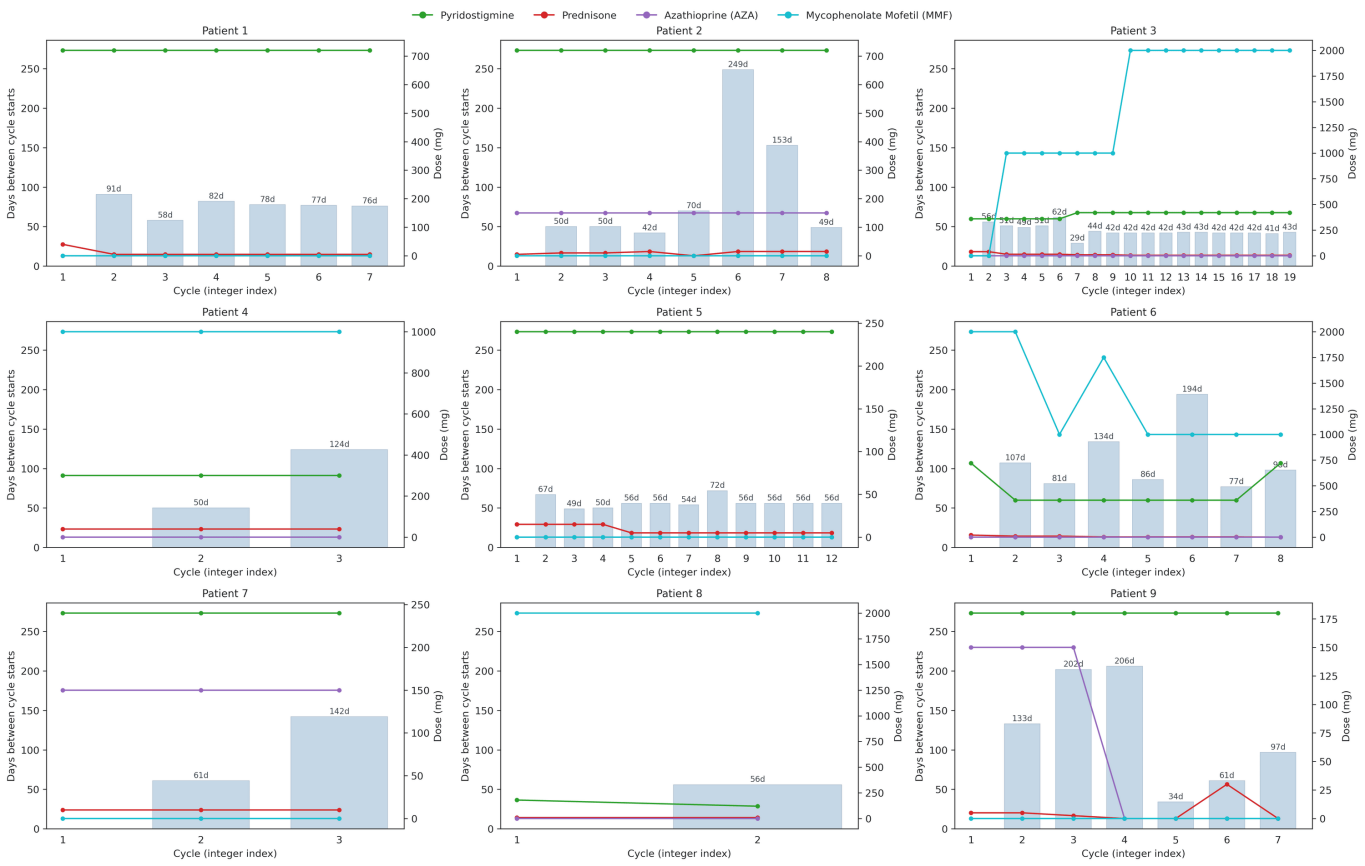
Overlay plots per patient showing days between cycle starts (bars) and doses of pyridostigmine, prednisone, AZA, and MMF at each cycle (lines with markers). Cycle 1 represents baseline; because there is no prior cycle, its interval spacing is undefined and omitted. For subsequent cycles, bars display the number of days since the previous cycle start. Doses are recorded at the start of each cycle (N = 9). AZA, azathioprine; MMF, mycophenolate mofetil.

Figure 4 presents individual trajectories for nine patients, depicting days between treatment cycles (bars) and concomitant medication doses recorded at the start of each cycle (lines for pyridostigmine, prednisone, AZA, and MMF). The number of cycles per patient ranged from 2 to 19. Across patients, both cycle intervals and medication dosing patterns were heterogeneous. Several patients showed relatively stable intervals and doses over time (e.g., Patient 5), whereas others exhibited marked fluctuations, including prolonged gaps between some cycles (e.g., Patient 2, cycle 6: 249 days; Patient 6, cycle 7: 194 days; Patient 9, cycle 4: 206 days). Dosing of immunosuppressants and pyridostigmine varied by patient, with some maintaining steady regimens and others undergoing substantial adjustments across cycles. The effect of concomitant medication use on cycle intervals was analyzed using within-patient fixed-effects models adjusting for cycle index (Table 3). While most medications showed no clear association with subsequent cycle length, higher AZA dosing was consistently linked to longer intervals. This may reflect that patients on higher AZA doses had more stable disease, allowing clinicians to extend infusion intervals safely, or that AZA provides sufficient immunosuppressive coverage to reduce the need for frequent cycles. These interpretations remain speculative given the small sample size.

**Table 3:** Regression results showing the association between baseline medication doses and subsequent efgartigimod infusion intervals. Positive coefficients indicate longer intervals in days. Fixed-effects models included patient indicators and cycle index with HC3 robust standard errors. Mixed-effects models included random patient intercepts. Medication predictors were scaled as follows: prednisone per 10 mg, pyridostigmine per 60 mg, AZA per 50 mg, and MMF per 250 mg. For each coefficient, we report the corresponding test statistic, defined as the estimated coefficient divided by its standard error (β/SE) (N = 9). AZA, azathioprine; MMF, mycophenolate mofetil.

Model	Predictor	Effect	95% CI	Test statistic	p-Value
Fixed-effects (next interval, days)	prednisone_per10	1.16	(-18.03, 20.36)	0.12	0.905
Fixed-effects (next interval, days)	pyridostigmine_per60	2.37	(-18.62, 23.36)	0.22	0.825
Fixed-effects (next interval, days)	aza_per50	41.31	(17.84, 64.77)	3.4	0.001
Fixed-effects (next interval, days)	mmf_per250	-5.59	(-12.92, 1.74)	-1.5	0.135
Fixed-effects (next interval, log-days)	prednisone_per10	0.07	(-0.12, 0.25)	0.72	0.474
Fixed-effects (next interval, log-days)	pyridostigmine_per60	0.02	(-0.22, 0.25)	0.14	0.887
Fixed-effects (next interval, log-days)	aza_per50	0.41	(0.16, 0.65)	3.3	0.001
Fixed-effects (next interval, log-days)	mmf_per250	-0.05	(-0.11, 0.01)	-1.5	0.12
Mixed-effects (next interval, log-days)	prednisone_per10	0.06	(-0.06, 0.18)	1.05	0.295
Mixed-effects (next interval, log-days)	pyridostigmine_per60	-0.01	(-0.08, 0.05)	-0.42	0.675
Mixed-effects (next interval, log-days)	aza_per50	0.28	(0.1, 0.46)	3.1	0.002
Mixed-effects (next interval, log-days)	mmf_per250	-0.01	(-0.07, 0.05)	-0.44	0.659

## Discussion

Treatment cycle intervals and patient outcomes

The principal findings of this study include a mean time of 47 days between treatment cycles and an average MG-ADL score of 8.56 at baseline and 5.29 at 12 months. The current infusion interval recommendation is once per week for four doses as one cycle, with future cycles administered according to clinical evaluation [23]. Our study found large variability in real-world dosing schedules, with some patients having much longer intervals between cycles, while others had shorter intervals followed by longer ones. This large variability in treatment cycle length may have impacted our patients’ treatment outcomes. However, it may also reflect the necessity of individualized treatment for MG [24].

Notably, in the ADAPT study, patients were required to worsen by at least 2 points on the MG-ADL before they were eligible for redosing. In contrast, in our clinical practice, the decision to redose was based on shared decision-making with the patient, usually at the first sign of any perceived symptom worsening, even if it did not meet the 2-point threshold. This more flexible, patient-centered approach allowed for earlier retreatment and may explain some of the differences in dosing intervals seen in our data. We are interested in investigating the cause of these dosing schedule fluctuations in future research.

Given the small sample size and single-center design, these observations should be considered preliminary. The wide variability in treatment intervals highlights the individualized nature of MG management rather than definitive evidence for optimal dosing schedules.

Efgartigimod efficacy in clinical trials

Efgartigimod has demonstrated efficacy in clinical trials. In a phase 2 study, Howard et al. found that 75% of patients receiving efgartigimod demonstrated rapid and long-lasting disease improvement [25]. In the phase 3 trial of the prior study, Howard et al. found that MG patients who used efgartigimod had 4.95 times higher odds of being MG-ADL responders (≥2 point MG-ADL improvement sustained for ≥4 weeks) compared to those receiving placebo [15]. Our study result of a mean 5.29 MG-ADL decrease from baseline is consistent with this.

Real-world effectiveness of efgartigimod

There is limited data on efgartigimod in real-world environments due to the recency of its FDA approval. Frangiamore et al. found that in a study of 19 real patients with MG, none were hospitalized while on efgartigimod. In contrast, eight of the 19 patients (42%) were hospitalized in the year before starting efgartigimod [26]. Additionally, Dionísio et al. found that 75% of patients had a mean reduction in the MG-ADL of ≥2 points in the first cycle, and this remained stable throughout the 12-month study [27].

These results, combined with ours, offer increasing evidence of the drug’s effectiveness. Our concomitant medication analysis provides additional insights. While baseline dosing of prednisone, pyridostigmine, and MMF was not clearly associated with cycle timing, higher AZA doses consistently predicted longer infusion intervals across statistical models. This suggests that specific immunosuppressant regimens, particularly AZA, may interact with clinical decision-making around redosing, although the small sample size limits definitive interpretation.

Emerging therapies and cost considerations

Several new therapies aside from efgartigimod have emerged in the last 10 years to treat MG. Eculizumab and ravulizumab (both complement inhibitors) are shown to be effective at treating refractory MG [17]. Zilucoplan was the first FDA-approved MG therapy involving daily self-injection [28]. Rozanolixizumab, like efgartigimod, is an FcRn and is the first FDA-approved treatment for anti-AChR and anti-MuSK antibody-positive MG [10]. Nipocalimab is also now available as an every-two-week intravenous FcRN inhibitor for anti-AChR and anti-MuSK antibody-positive MG.

A potential hindrance to the use of these medications is the cost. These medications are all quite expensive, and insurance coverage is variable [29]. For example, efgartigimod can cost up to $450,000 annually when dosed 13 cycles per year [6]. Lien et al. found that the incremental cost-effective ratios (ICERs; cost per quality-adjusted life-year (QALY) gained) for eculizumab and efgartigimod were $3,310,000/QALY gained and $1,959,000/QALY gained, respectively. The commonly accepted willingness-to-pay (WTP) threshold is $200,000/QALY gained, so these ratios for eculizumab and efgartigimod are significantly above this threshold [6]. The same study found that eculizumab would need to be discounted by 93.01% and efgartigimod by 88.34% in order to be cost-effective [29]. However, a different Canadian study found that over the lifetime, efgartigimod provides greater benefit at lower costs than chronic IVIg for AChR-Ab+ gMG patients [7]. Regardless of the treatment used, cost may be a major factor in patients’ ability to receive any of the newer MG medications.

Safety profile of efgartigimod

While our study did not systematically collect adverse event data, the literature suggests that efgartigimod is generally well tolerated. Common short-term side effects include headaches, upper respiratory tract infections, urinary tract infections, and mild infusion reactions [30]. Long-term safety data remain limited due to the recency of FDA approval; however, extended follow-up in clinical trials and early real-world reports indicate a low risk of serious adverse events. No cumulative toxicity or organ-specific toxicity has been reported to date. Future studies should aim to monitor long-term adverse events, including potential immunologic effects, infection risk, and rare hematologic or hepatic complications, to better characterize the full safety and tolerability profile of efgartigimod in chronic use.

Study limitations and future directions

There were a few limitations in our study. First, our sample size was small, with only nine patients. While these patients provided valuable data on efgartigimod, a larger sample size would yield more robust data. Our data show wide variability in dosing schedules. A larger sample size would help provide more information on whether this is a consistent finding or simply a unique feature among the nine patients in the sample. If widely variable dosing schedules still provide a sustained response, this may be a unique and desirable feature of efgartigimod, allowing for highly individualized treatment plans. A study that includes patients from multiple hospital systems would be beneficial in order to achieve a larger sample size.

Additionally, this study looks at a maximum follow-up time of 12 months after initiation of therapy. It would be valuable to get longer-term follow-up information on efgartigimod, which has proved challenging due to the recency of the drug’s approval. Finally, given the retrospective nature of this study, it was difficult to collect MG-ADL scores at a consistent time period between all patients included in the study. In the future, it may be more fitting to perform similar studies as prospective rather than retrospective in order to ensure complete and accurate documentation. As efgartigimod becomes more widely used for MG treatment, we recommend increased communication between infusion centers and medical practices to ensure accuracy.

## Conclusions

Efgartigimod was associated with preliminary improvements in MG-ADL scores in this small cohort, with a mean 5.29-point decrease over 12 months. Due to the small sample size and single-center design, these results are hypothesis-generating rather than definitive. Despite variability in cycle intervals, outcomes were consistent with trial data, supporting the need for further investigation into flexible, patient-centered dosing schedules.

Analysis of concomitant medications showed no clear association between prednisone, pyridostigmine, or MMF dosing and cycle length, but higher AZA dosing was linked to longer infusion intervals, potentially reflecting stable disease or clinician preference, but requiring confirmation in larger studies.

Future studies with larger, prospective cohorts and longer follow-up are necessary to clarify the durability of response, define safe and effective approaches to cycle timing, and determine the role of concomitant therapies, particularly AZA. Economic considerations remain important, as the high cost of newer agents poses challenges to broader adoption.
